# Scoliosis short-term rehabilitation (SSTR) according to 'Best Practice' standards - are the results repeatable?

**DOI:** 10.1186/1748-7161-7-1

**Published:** 2012-01-17

**Authors:** Maksym Borysov, Artem Borysov

**Affiliations:** 1 "Biotechnika" Rehabilitation Services, Moskovsky prospekt 197, Kharkov (61037), Ukraine

## Abstract

Claims have been made in a pilot study that a new form of short-term rehabilitation according to 'Best Practice' standards would change signs and symptoms of patients with scoliosis in the short-term. Aim of this study is to repeat the study published 2010 with a larger sample of patients using the same protocol. Both authors have undergone training in this special approach to scoliosis rehabilitation in 2010.

**Materials and methods:**

34 patients with Adolescent Idiopathic Scoliosis (AIS), 32 girls and 2 boys, average age 13.7 years and an average Cobb angle of 28.7 degrees (21-43 degrees) underwent Scoliosis Short-Term Rehabilitation (SSTR) of seven days. Two days with an intensity of 3 × 90 min sessions/day, and five days with an intensity of 2 × 60 min sessions/day. Angle of trunk rotation (ATR) was measured before and after the time of treatment as well as the active correctability of the ATR after the programme as it has been done in the pilot investigation. Additionally to that, we also recorded the changes in Vital Capacity (VC) before and after the programme.

**Results:**

ATR was reduced significantly from 11,5 degrees to 8,4 degrees, the active correctability as measured with the Scoliometer (TM) was also reduced significantly from the ATR after treatment 8,9 degrees to 6,5 degrees in the patients with thoracic curves. VC improved significantly (P < 0,05) from 2073 ml to 2326 ml.

**Discussion:**

The results achieved in the pilot investigation published previously are repeatable. The deformity of the trunk can be reduced significantly after SSTR. During the pilot study VC was not investigated. In our study VC improved significantly. Therefore, also shorter rehabilitation times with an appropriate programme seem to be able to change signs and symptoms of a patient with scoliosis. Like the out-patient Schroth programme as described in a study from Turkey, the SSTR provides benefits leading to an improvement of the condition.

**Conclusion:**

Out-patient rehabilitation following the Scoliologic (TM) 'Best Practice' standards seems to provide an improvement of signs and symptoms of scoliosis patients in this study using a pre-/post prospective design. The results of the pilot study therefore seem to be repeatable.

## Background

The original Schroth programme [1] in the conservative treatment of patients with scoliosis was designed for curves exceeding 70° or 80°, while the indication for physiotherapy alone as seen today, is significantly different [2]. The original programme has been applied over periods of three months or even longer [3], later in the 1960's and 1970's the patients were treated with an in-patient programme of six weeks [3].

Recent studies have shown that the treatment time can be reduced [4] and that the original Schroth programme can be improved by adding certain modules of treatment [5].

Independently in a cohort study from Turkey, the Schroth programme has been investigated and the authors found significant improvements of all signs and symptoms of a scoliosis over a certain time of treatment [6].

The over-all evidence for physical therapy in the treatment of scoliosis on the other hand may be weak with respect to patient samples with a clear indication of treatment [7], however, at least one prospective controlled study is available in a patient sample with the majority of patients at risk for being progressive [8].

Recently, a paper has been published with a small sample of patients having undergone a new five-day short-term rehabilitation programme [9]. Claims have been made from this study that signs and symptoms of a scoliosis can be improved with this short programme adapted to latest evidence. The sample was very small including only nine patients with scoliosis with a wide range of curvature sizes.

Purpose of our investigation was to replicate this study with our own patients and materials in order to see as to whether the results of the pilot study can be achieved independently at our centre. Both authors have been taking part in the first B-Level (professional level) course to learn the application of the programme both theoretically and practically. Back in Kharkov, Ukraine, we gained some practical experience with the programme initially and then we began with this investigation.

## Materials and methods

Thirty-four patients with Adolescent Idiopathic Scoliosis (AIS), 32 girls and 2 boys, average age 13,7 years and an average Cobb angle of 28,7° underwent Scoliosis Short-Term Rehabilitation (SSTR) for seven days. All patients had a brace and they came to our centre for brace renewal as it was outgrown. So the SSTR program was performed while the patients were waiting for the new brace. During the treatment time no brace was worn.

The treatment was performed in small groups of 2 patients with similar curve pattern.

The sample consisted of 20 double curves, 13 thoracic single curve patterns and 3 single lumbar/thoracolumbar curve patterns. According to the augmented Lehnert-Schroth classification [3] we had the following distribution of curve patterns: 20 patients with a 4C pattern, 4 patients with a 3CN pattern, 7 patients with a 3CH pattern, 1 patient with a 3CTL pattern, 2 patients with a 4CL and 1 patient with a 4CTL pattern. The classification has been described before [3] and can be seen in Figure 26 from that paper.

The programme consisted of two days with an intensity of 3 × 90 min sessions/day, and five days with an intensity of 2 × 60 min sessions/day. The total treatment time compares well to the pilot investigation [9].

The schedule used for the treatment of our patients are documented in Additional file 1. Recently the programme has been reduced to 3 days, only (Additional file 2).

Angle of trunk rotation (ATR) was measured with a Scoliometer™ in forward bending from upright stance before and after the time of treatment as well as the active correctability of the ATR in the thoracic region after the programme as has been done in the pilot investigation. Additionally to that we also recorded the changes in Vital capacity (VC) before and after the programme (technical error < 8%).

**Figure 1:**
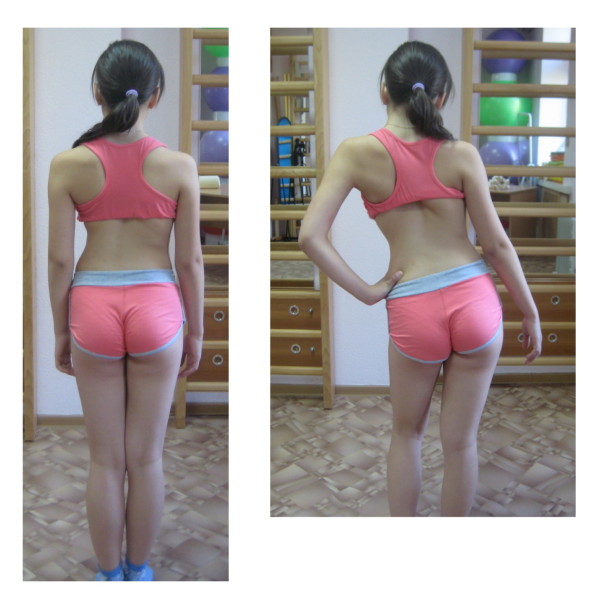
**Patient with right thoracic scoliosis (functional 3-curve pattern as seen on the *left*) performing the correction of ADL in upright position (*right*)**.

**Figure 2:**
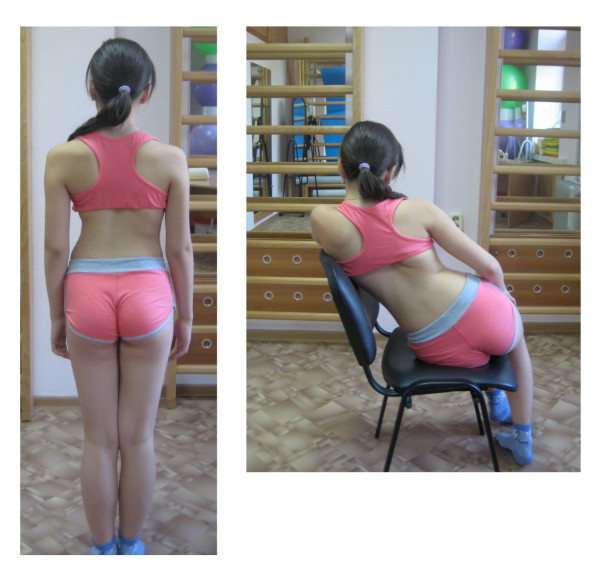
**Patient with right thoracic scoliosis (functional 3-curve pattern as seen on the *left*) performing the correction of ADL in sitting position (*right*)**.

**Figure 3:**
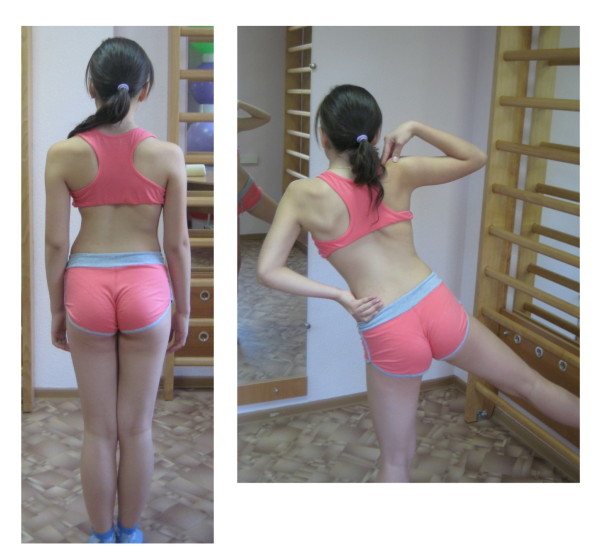
**Patient with right thoracic scoliosis (functional 3-curve pattern as seen on the *left*) performing the Muscle cylinder exercise according to the new ‚Power Schroth' principles (*right*)**. The corrected head alignment is not yet achieved and the correction of the sagittal profile can also be improved. Frontal plane correction is obviously visible.

**Figure 4:**
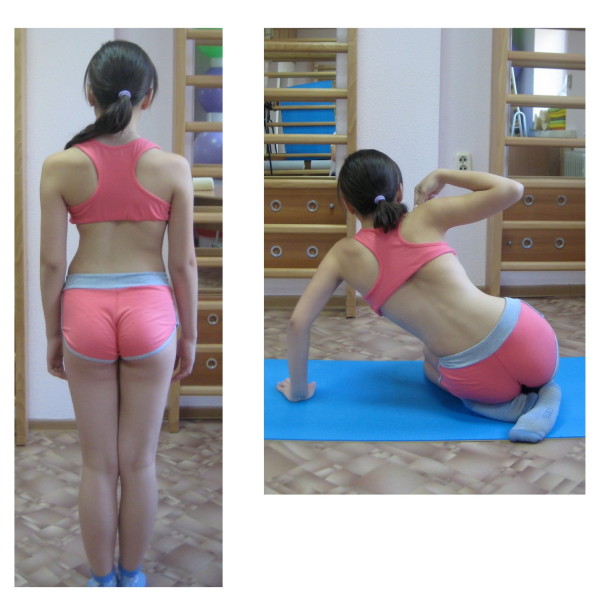
**Patient with right thoracic scoliosis (functional 3-curve pattern as seen on the *left*) performing the exercise ‚Frog at the pond' according to the new ‚Power Schroth' principles (*right*)**. The corrected head alignment is not yet achieved but correction of the sagittal profile is already visible.

**Figure 5:**
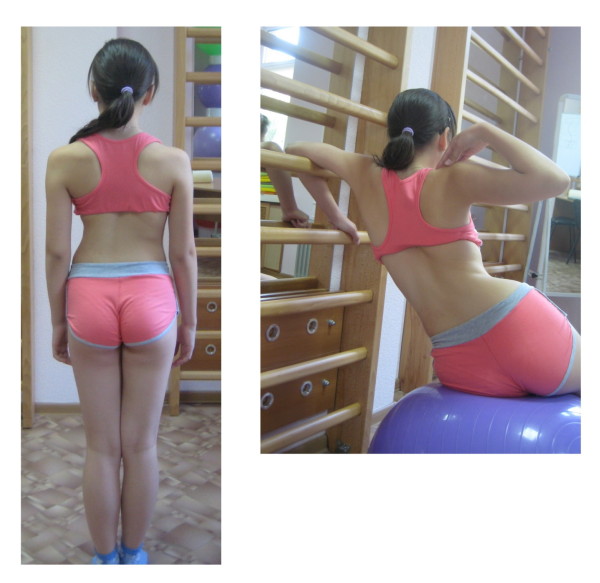
**Patient with right thoracic scoliosis (functional 3-curve pattern as seen on the *left*) performing the ‚Door handle exercise' according to the new ‚Power Schroth' principles (*right*)**. The corrected head alignment is not yet achieved but correction of the sagittal profil is already visible.

The programme (described more deeply in Additional file 3) consisted of (1) correction of the sagittal profile, (2) corrections of the activities of daily living (ADL; see also Figure 1 and 2), (3) 3D-made easy exercises and (4) New Power Schroth exercises (Figure 3, 4, 5). 3D-made easy exercises and New Power Schroth exercises are enforced by 'Rotational Breathing' exercises [1] and therefore may improve VC. Stabilisation with trunk muscle tension during exspiration in these two programs is of major importance as well as is in the original Schroth programme.

The programmes were structured as follows: physio-logic^® ^and ADL modules-30 min., '3D-made-easy' exercises 5 sets repeated 10 times, 'New Power Schroth' exercises 5 sets repeated 10 times as well.

The differences between the ATR values before and after the programme were evaluated using the t-Test, as were the ATR values after the programme in the 31 patients with thoracic curvatures where we measured the active autocorrectability (autocorrective movement as measured with a Scoliometer™ in forward bending from upright stance) each individual patient was able to achieve. The three patients with the single lumbar/thoracolumbar curve patterns were not undergoing the measurements for 'autocorrectability ' as like in the initial pilot investigation [9] only patients with thoracic curvatures where measured.

Additionally, we also evaluated the VC before and after the programme using the t-Test.

Written informed consent has been achieved from the patient visible on the pictures.

## Results

ATR was reduced significantly from 11,5° before the initiation of the programme to 8,4° at the end of the programme (p < 0,001), the active correctability as measured with the Scoliometer™ was also reduced significantly from the ATR after treatment 8,9° to 6,5° (p > 0,001). VC also improved significantly (p < 0,05) from 2073 ml to 2326 ml.

The ATR reductions did not correlate with curvature magnitude or with the age of the patients treated.

The individual results obtained for each single patient are documented in the table (Additional file 4).

## Discussion

The results of the pilot study published in 2010 [9] were shown to be repeatable. The programme is easy to learn and can be applied everywhere. The deformity obviously can, at least temporarily, be reduced significantly with the help of specific pattern dependent correction exercises. Additionally, an improvement of VC is possible, which has not been investigated in the pilot study. Improvements of VC were achieved with the original Schroth programme on an in-patient basis [10]. We were able to achieve comparable effects within a shorter period of time.

In the light of the latest developments in bracing, physiotherapy must be seen as an 'add on' treatment during the pubertal growth spurt, while bracing has to be acknowledged as the primary treatment of patients with scoliosis during growth [11-15]. Although the results of the latest bracing technology seems promising for the patients at actual risk for being progressive [16-18], physiotherapy is a worthwhile alternative to spinal fusion surgery in patients not at actual risk, considering that spinal fusion surgery will not change signs and symptoms of a scoliosis [11,19,20].

Although there is no evidence that the improvements of trunk deformity as measured with the help of the Scoliometer^® ^are stable in the long-term, there also is no evidence that the reduction of trunk deformity is stable after surgery [20].

We are well aware of the fact that mid to long-term studies are needed in order to establish a body of evidence for the use of specific exercises [6-10,21,22], however these preliminary results of a simple and effective approach seem promising.

As has been shown, the programme used is repeatable and therefore we encourage physical therapists to take advantage of the programme, which is described at large in two books [14,15].

At this stage, there are no mid- or long-term results available, so the results obtained from this study must be regarded as being preliminary. Further long-term results are needed to validate these findings.

As most of our patients come from the Ukraine we will follow-up the patient sample as described prospectively in order to gain mid- and long-term effects in a few years.

## Conclusions

The SSTR programme seems to be reproducible with respect to the methodology and the results. The SSTR programme is an alternative for patients not at actual risk for being progressive. Patients during the pubertal growth spurt should be braced with high correction braces initially whenever indicated. The SSTR programme is based on a certain classification of curve patterns and therefore is easily repeatable after a short time of training within the course programme provided (Additional file 2).

## Competing interests

The authors declare that they have no competing interests.

## Authors' contributions

MB and AB contributed equally in study design, patient acquisition, patient management during treatment, manuscript writing and statistical analysis. Both authors read and approved the final manuscript.

## Supplementary Material

Additional file 1 **Description of the schedule of the patients undergoing SSTR at our centre**. Click here for file

Additional file 2 **Description of the course programme provided by the Scoliologic™ Best Practice academy, showing that the SSTR has been reduced to a program of 3 days, only**. Click here for file

Additional file 3 **Description of the basic principles used within the Scoliologic™ Best Practice program (Chapter IV from the 4^th ^edition of **[15]**with kind permission by Dr. HR Weiss)**. Click here for file

Additional file 4 **Table with the individual results obtained for each single patient (raw data of each patient)**. Click here for file
