# Supplementary material for: Scoliosis short-term rehabilitation (SSTR) according to 'Best Practice' standards - are the results repeatable?
Source: Scoliosis. 2012 Jan 17;7:1. doi: 10.1186/1748-7161-7-1 (PMC3292465; doi:10.1186/1748-7161-7-1)
Supplement: Additional file 1 — Description of the schedule of the patients undergoing SSTR at our centre. [file 1748-7161-7-1-S1.PDF]

SSTR according to the Scoliologic™ 'Best Practice' standard as performed in the Ukraine

The programme was structured as follows:

- physio-logic® and
- ADL modules – 30 min.,
- '3D-made-easy' exercises 5 sets repeated 10 times,
- 'New Power Schroth' exercises 5 sets repeated 10 times as well.

The programme started with physio-logic™ exercises and included also 'experiencial learning' sessions to enable the patients to acquire certain skills by themselves without the help – only under supervision – of the therapist. The basic principle of the concept is to always repeat the whole programme before a new part of the programme structure is tought.

The schedule is described in the following:

Day 1:

- Welcome – physio-logic™ 90 min
- Experiencial learning: curve patterns & physical examination 90 min
- Scoliosis patterns and correction of ADL 90 min

Day 2:

- ADL & Walking 90 min
- Experiencial learning: Systematic self correction 90 min
- Systematic self correction & 3D-made-easy 90 min

Day 3:

- Whole programme + 3D-made-easy 60 min
- Whole programme + New Power Schroth I (50 times exercise) 60 min

Day 4:

- Experiencial learning (Repetition + 3D correction during walk) 60 min
- Whole programme + New Power Schroth II (+ Door handle exercise) 60 min

Day 5:

- Whole programme + New Power Schroth III (+ Muscle cylinder exercise) 60 min
- Whole programme + New Power Schroth IV (+ Frog at the pond exercise) 60 min

Day 6:

- Whole programme + New Power Schroth V (+ hip lifting exercise) 60 min
- Whole programme + 3D correction during walk (highest level) 60 min

Day 7:

- Whole programme 60 min
- Whole programme + Evaluation of patients' knowledge 60 min

Literature:

[1] Weiss HR: Befundgerechte Physiotherapie bei Skoliose, Pflaum Verlag, Munich, 2011

[2] Weiss HR: Best Practice in conservative scoliosis care, 4th edition, Pflaum Verlag, Munich, 2012
