# Supplementary material for: Scoliosis short-term rehabilitation (SSTR) according to 'Best Practice' standards - are the results repeatable?
Source: Scoliosis. 2012 Jan 17;7:1. doi: 10.1186/1748-7161-7-1 (PMC3292465; doi:10.1186/1748-7161-7-1)
Supplement: Additional file 2 — Description of the course programme provided by the Scoliologic™ Best Practice academy, showing that the SSTR has been reduced to a program of 3 days, only. [file 1748-7161-7-1-S2.PDF]

August | 11

The ScoliOlogiC™ Best Practice course program

Hans-Rudolf Weiss, MD

Short description of the A-, B- and I-level curriculum including an outlook on a more individualized bracing seminar.

© ScoliOlogiC™ Best Practice Academy
Orthopedic Rehabilitation Services
Gesundheitsforum Nahetal
Alzeyer Str. 23; 55457 Gensingen, Germany

The ScoliOlogiC™ *Best Practice* course program

The official Scoliologic® course program (I, A and B) is designed for Physical Therapists, Physicians and Chiropractors to allow the acquisition of the skills necessary to guide their scoliosis patients professionally, to diagnose curve patterns and to apply pattern specific exercises for the treatment of scoliosis.

Attendees of the A-Level courses already are able to treat their patients properly, however treatment security increases drastically when professionals have attended the B-Level course with the experience of the treatment of patients with different curve patterns as well.
In conservative treatment of scoliosis during growth brace treatment has to be regarded as the primary treatment. Therefore, it is highly advisible to implement the Scoliologic® ‚Best Practice' bracing program as well.
The Scoliologic® ‚Best Practice' is not compatible with low correction bracing or with the use of soft braces! There is no evidence from independent studies that soft braces do work. In the contrary, during the pubertal growth spurt the application of these braces is of very limited benefit as has been shown in two independent prospective controlled studies, one of them randomized.
Therefore the implementation of the Scoliologic® ‚Best Practice' bracing program is highly advisable.

Scoliologic® A- and B-Level course

Therapists only (A-Level course 2 days)*:

Saturday:

- Lectures (Conservative management of patients with spinal deformities – physiotherapy – braces – scientific evidence)
- Physio-logic™ (Standing, sitting and while walking)
- Experiencial learning session (Workout of curve patterns)
- Correction of the Activities of daily living (ADL) in standing and sitting
- 3D-made-easy exercises + first ideas of Schroth

Sunday:

- Lectures (Indications, curve patterns, deformities of different etiologies)
- Whole 1st. Day program + First Schroth exercise
- Whole program + 2nd. And 3rd. Schroth exercise
- Whole program + 4th. And 5th. Schroth exercise
- Examination

Therapists together with patients (B-Level):

Monday:

- Welcome – physio-logic™ 90 min
- Experiencial learning: curve patterns & physical examination 90 min
- Scoliosis patterns and correction of ADL 90 min
- ADL & Walking 90 min

Tuesday:

- Experiencial learning: Systematic self correction 90 min
- Systematic self correction & 3D-made-easy 90 min
- Whole program + 3D-made-easy 90 min
- Rehabilitation of walk 90 min

Wednesday:

- Whole program + Schroth I 90min
- Experiencial learning (Repetition) 90 min
- Whole program + Schroth II & III 90 min
- Whole program + Schroth IV & V 90 min

The Scoliologic® ‚Best Practice' bracing program

Orthopedic technicians are advised to follow an official I-course at first. After that individual arrangements for a Scoliologic® brace course can be made. It is advisable to calculate 7 workdays to follow the brace manufacturing, adjustment, application and professional brace checks. Within this time a certain amount of experience can be achieved so as to allow to start bracing at a certain professional level.
Official course language is English but can be offered in Russian language as well. Whenever translation to other languages is necessary the time schedule cannot be kept as planned, therefore the schedule must be considered as being 30% longer.

Scoliologic® Information course

Therapists only (I-course 1.5 days):

Saturday:

- 2 Lectures (Conservative management of patients with spinal deformities – physiotherapy – braces – scientific evidence)
- Patient demonstration

Sunday:

- 2 Lectures (Indications, curve patterns, deformities of different etiologies)
- Questions and answers

* A *one day* A-Level course containing the practical parts of the regular A-Level course can be made available to participants who have undergone the official Information (I-) course before.

© ScoliOlogiC™ Best Practice Academy
