# Supplementary material for: Scoliosis short-term rehabilitation (SSTR) according to 'Best Practice' standards - are the results repeatable?
Source: Scoliosis. 2012 Jan 17;7:1. doi: 10.1186/1748-7161-7-1 (PMC3292465; doi:10.1186/1748-7161-7-1)
Supplement: Additional file 3 — Description of the basic principles used within the Scoliologic™ Best Practice program (Chapter IV from the 4th edition of [15]with kind permission by Dr. HR Weiss). [file 1748-7161-7-1-S3.PDF]

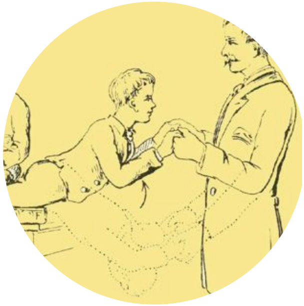

IV. Physiotherapy

The application of physiotherapy to date is discussed with contrary points of view in international literature.

In a systematic review Fusco et al. [1] found one randomized controlled study from China supporting the effectiveness of physical exercises however this included a sample of more mature girls not at risk for being progressive. The authors found the quality of the studies to be very poor: Many studies [2,3,4,5] were uncontrolled, and involved only one evaluation of the outcome measured before and after the intervention. This kind of design produces results that are impossible to interpret since it is not possible to conclude reasonably that the improvement observed was causally determined by the intervention. The positive change could have occurred naturally or might have been the result of other aspects of therapy being conducted contemporaneously [6].

Three out of the six controlled studies were prospective [7,8,9], but two of these used a historical control group [7,8]: in such cases, there may be many factors, apart from the experimental intervention, in which the experimental and the control groups differ from one another. Of the four studies that did have a concurrent control group [9,10,11,12], only one [11] specified the allocation criterion, which was patient preference. The method of recruitment was described in only few studies [7,8]. None attempted to obtain a blinded assessment of the outcome, even though such an assessment should always be introduced as a means of limiting the possibility of detection bias, when it is impossible to ensure the patients unawareness of those administering the interventions [13].

In the USA as well as in Great Britain there has been only a small amount of clinical research to test the efficacy of proactive, physiotherapy-based methods to treat IS in early stages when curvatures are mild [7,8,14,15,16,17].

The rationale to justify lack of early intervention is that in natural history surveys, small curvatures of <15 degrees often remain stable and may even improve while the child is under observation [2,18]. Unfortunately, 'natural history' studies which underlie our existing knowledge base are compromised by the fact that most have included an unknown and undescribed proportion of patients who received physiotherapy including exercises and manipulation [19,20,21,22,23,24,25,26,27]. Because the impact of such treatment has been ignored, the possibility that some curvatures stabilized or improved in response to conservative therapy cannot be ruled out [28]. It is possible that this situation reflects a long-standing bias against exercise-based therapies in the treatment of IS in English speaking countries, especially the U.S. This bias is reflected in statements such as:

Modified from:
Hawes M.C.: The use of exercises in the treatment of scoliosis: an evidence-based critical review of the literature.
Pediatr Rehabil. 2003 Jul-Dec;6(3-4):171-82.

"To sum up the indications for an exercise program, you can prescribe it if you wish, as long as you understand that exercises only treat the psyches of the parents and help the muscle coordination of certain poorly muscled children, who are overweight and underexercised" [29].

This largely reflects the opinion of the surgical community. Physical Medicine and rehabilitation physicians, physiotherapists and other conservative specialists involved in scoliosis management will look at their field another way. Are they biased? Is their experience different? Many questions arise and we may ask ourselves: "What is right? Is the randomised controlled trial the only study design to prove a hypothesis? Is the Cobb angle the most important sign to be treated?"

Scoliosis is a three-dimensional deformity in which the spine deviates from its normal sagittal and coronal positions in the upright human posture, and becomes fixed in this unbalanced posture [30,31,32,33]. The mechanical imbalance inherent in scoliosis, irrespective of its cause, results in asymmetric loading which constitutes a 'vicious cycle' with an inevitable tendency to worsen with time [34,35,36]. In fact, most cases of scoliosis do continue to progress throughout the life of the patient [2,20,22,24,37,38,39]. Symptoms that occur in association with scoliosis include pain [20,24,25,26,40,41,42,43,44,45, 46,47,48,49,50,51] and psychological distress [2,52,53,54,55,56,57,58,59,60,61,62,63]. In curvatures involving the thoracic spine, reduced chest wall mobility and impaired chest wall expansion occur as a secondary effect of reduced spinal flexibility [64,65,66]. Reduced chest wall expansion causes restrictive lung dysfunction which is proportional to magnitude of curvature, and death by cardiac or respiratory failure can occur when the Cobb angle is >70 degrees [51,67,68,69,70,71]. Even mild or moderate cases which remain stable are associated with pulmonary dysfunctions including reduced vital capacity, reduced exercise capacity, and additionally with recurrent respiratory infections [72,73,74,75,76,77,78,79,80,81,82,83]. Impaired respiratory function in scoliosis generally develops gradually over time and therefore is 'symptomless' because patients adapt to reduced function and remain unaware of their limitations; therefore cardiopulmonary failure may occur unexpectedly in response to the onset of respiratory infection [67,69,84,85,86,87].

Case studies have demonstrated that measurable positive changes in the signs and symptoms of IS are correlated with conservative management [4,6,88,89]. Among >800 patients, nearly every case revealed a small but significant improvement in chest expansion and a 14-19% improvement in VC after conservative treatment [89]. Among 794 adult patients with severe scoliosis, 55% exhibited at least one sign of right ventricular strain at admission, and by the end only 12% exhibited signs of right ventricular impairment; VC improved by 250 ml in the same population [90]. Among 107 patients the mean Cobb angle decreased from 43 to 39 degrees, with improvements of up to 20 degrees in indivi-

dual patients after conservative treatment [5]. Studies also have demonstrated significant improvement in pain [36,61,62] and psychological distress [87,88] in response to conservative treatment. Results of a preliminary study were consistent with the possibility that the incidence of progression among 181 patients treated with physiotherapy during the late 1980's was significantly less than the incidence that would be expected, based on natural history surveys [6].
Another study to test the hypothesis that physiotherapy-based intervention can reduce the incidence of progression in children with IS was done recently [9]. The content was a follow-up of the outcome of two prospective studies using the incidence of progression ($\geq$ 5°) as the outcome parameter, in treated and untreated patient groups matched by age, sex, and degree of curvature at diagnosis. A six-week intensive rehabilitation program offering patient-specific physiotherapy including intensive therapist-assisted exercise in diagnosis-matched groups was the method of treatment. The incidence of progression in groups of untreated patients ranged from 1.5-fold (71.2% vs 46.7%) to 2.9-fold (55.8% vs 19.2%) higher than in groups of patients treated with conservative treatment, even when the groups treated conservatively included patients with more severe curvatures. Statistically, the differences were highly significant. The results of this study indicate that a supervised program of exercise-based therapies can reduce the incidence of progression in children with IS.
Therefore there seems to be good reasons to assume that physical exercises at least in the environment of an in-patient program have a beneficial input on the signs and symptoms from which patients with AIS suffer.

Physiotherapists have to ask themselves what aims they should follow. In the SOSORT consensus paper [91] many aims of exercise treatment have been proposed.
The question arises whether to address all changes ever reported or try to determine real key points to develop a common standard of treatment. If the muscular and neurologic changes found are secondary abnormalities, they will automatically be changed by postural correction [92].
Scoliosis is a 3-D deformity of the spine so we will need "Autocorrection in 3-D". We cannot correct the spine 24 hours / day by exercising 30 minutes. So it might be reasonable to add "Ergonomy" to integrate the corrected postural stereotype into activities of daily living (ADL).
We have to acknowledge that "general health" is reduced in operated patients with AIS and also in patients treated conservatively [93]. This might be due to deficits of respiratory capacity [72-85]. Respiratory education, though, will be an important issue to be addressed by physiotherapists. Respiratory education may improve 3-D correction [94] and vital capacity as well [89].

If we agree that "Autocorrection in 3-D" is the major aim we can of course integrate also correction exercises in one plane like the "Side Shift" or "Sagittal Realignment Exercises" into our program, however these will not have to be named as singular aims per se if they serve our main aim in the end.
We will have to discuss whether to leave out aims serving only the secondary consequences of Idiopathic Scoliosis like coordination, equilibrium, general motor capacity, muscular endurance, muscular strength and neuromotor control that may be addressed automatically by autocorrection in 3-D.
If we agree that "autoelongation" might bear the risk of deteriorating the sagittal alignment and if we agree that "stabilisation" has to be integrated into autocorrection in 3-D, we could sum up our aims of treatment as follows: autocorrection in 3-D, training of ADL and respiratory care.

Autocorrection in 3-D seems to be the main aim of physiotherapy. That means we will have to train our patients to be able to perform those exercises on their own, if possible also during daily activities. However the term "Autocorrection in 3-D" is not yet defined clearly. In the Lyonaise program corrections are usually performed to reach a symmetric appearance while in the Schroth program a functional overcorrection in 3-D is the aim with the help of a special breathing technique and with the help of postural reflexes as well. If we follow the concept of growth modulation [95] maximum postural corrections should be performed in order to achieve the best possible outcome (Fig. 12.). Indeed the results gained with the help of the Schroth program seem to show the deepest impact with respect to signs and symptoms of scoliosis [88], however the planned SOSORT multicenter study will enlighten many open questions regarding this topic in the future.

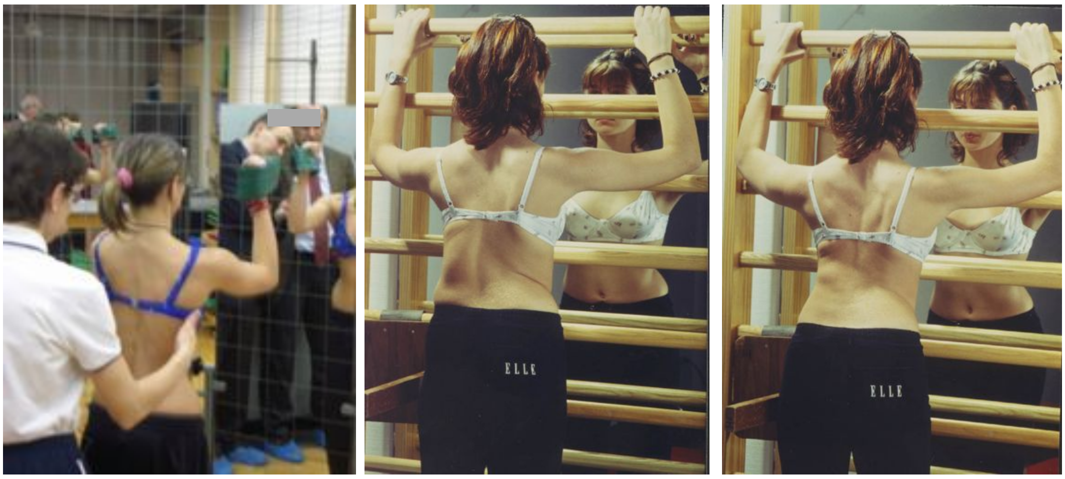

Fig. 12. *Left: Exercise in the SEAS setting. Parts of the 3D correction achieved may be lost again while exercising different parts of the trunk.*

Right: 3D postural correction is clearly visible using the original Schroth technique (right) when compared to the starting position: middle

The physio-logic® program

Thoracic flatback has been assumed to be the triggering factor for thoracic Idiopathic Scoliosis [96-100]. So if coupled rotation and lateral deviation of the spine are secondary patterns of deformity in the development of Idiopathic Scoliosis (IS), it should be possible to correct or improve scoliosis by the application of sagittal forces.
Flatback seems to be a major problem in the treatment of patients with idiopathic scoliosis using braces. It has been demonstrated that flatback may be increased instead of corrected using several bracing concepts [101]. There are also other studies to support especially the Boston brace to reduce sagittal curvatures of the spine and in a Pub Med search no study has been found to support the opposite. Boston braces, the Charleston bending brace and most of the other types of scoliosis braces until now have not had pressure points to address the sagittal profile. Only with the original Chêneau brace have pressure points been introduced for the correction of thoracic hypokyphosis [102].

Physiotherapy programs so far mainly address the lateral deformity of scoliosis [103-106], a few aim at the correction of rotation [4,11,12,107] and even fewer address the sagittal profile [108-110]. Prior to 1992 Negrini stated that the sagittal deformation is also important to correct with the help of exercises [111]. In some programs flatback is addressed extensively, however those programs failed to show good results [112]. All patients in the treatment program described in this article, worsened within one year of regular treatment. Therefore it would appear unreasonable to mobilize the scoliotic spine into a global kyphosis including the lumbar spine.

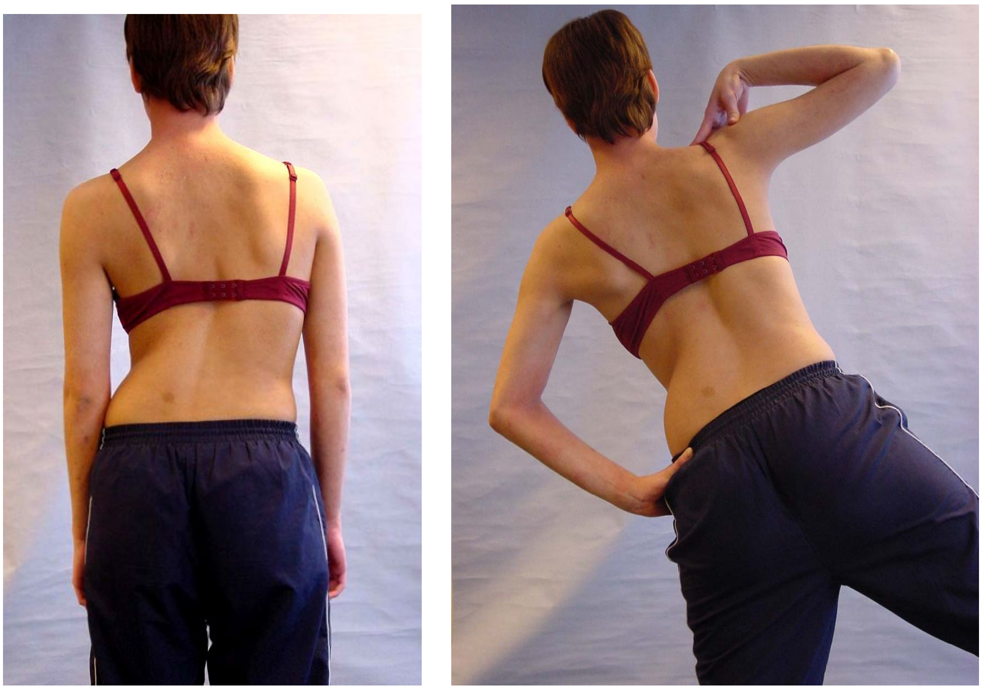

Fig. 13. *Although performing the Schroth rotatory breathing exercise flatback is clearly visible. Therefore it seems reasonable to increase efforts to correct scoliosis in the sagittal plane as well. This now is possible when using the "New Power Schroth" Method.*

In the Schroth program thoracic kyphosis is addressed by performing a special breathing technique, however in the exercising position the flatback is clearly visible (Fig.13.). The results of the Schroth concept seem to be quite good and show that progression can be prevented in many cases [6,9,113-115].

So the Schroth program has developed to be the gold standard of physiotherapy for the treatment of scoliosis of larger degrees in many countries as well as for in-patient rehabilitation [18].

Meanwhile there is evidence that correction forces applied in the sagittal plane are also able to correct the scoliotic deformity in the coronal and frontal planes [101]. In an experimental study comparing the short term effect of two different braces as can be measured with the help of surface topography, it has been shown that sagittal correction forces lead to similar short term corrections, as the 3-D correction braces, which are at the moment the gold standard in many European countries.

Although the Schroth method addresses the sagittal plane in the long term [109], the results achieved in the experimental study suggest that we should improve excellence in scoliosis rehabilitation by the implementation of exercises to improve the correction of the sagittal deformity in scoliosis patients. We developed an exercise program aiming at a physiologic sagittal profile either to be applied to the program at our centre or to replace certain exercises or exercising positions in use. The exercises of this program all have the same basic principle to increase and so improve lumbar lordosis at L2 level and lower thoracic kyphosis and are called physio-logic exercises. The results obtained in an age, sex, curve pattern and Cobb-degree matched controlled study were consistent with the hypothesis that the application of physio-logic exercises improves the short term outcome of in-patient rehabilitation [116].

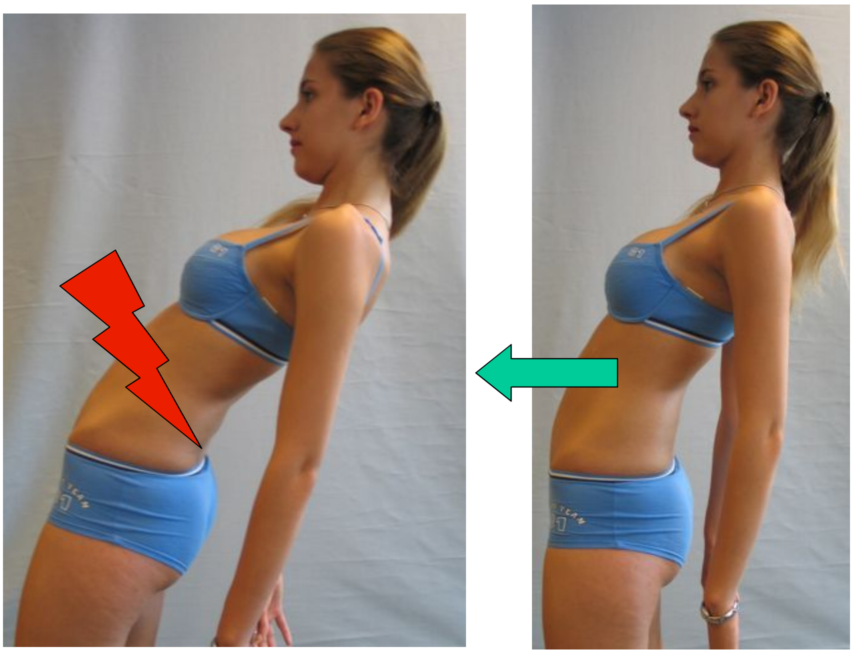

Fig. 14. *Simply reclining the trunk leads to an increased thoracic kyphosis, and also to increased stress in the lumbosacral region. This can be prevented by ventralizing the lower ribs in order to increase lordosis at the L2 level (right); [modified from 116].*

Description of the physio-logic® exercise program

Within the physio-logic® exercise program we provide:

- Symmetric mobilizing exercises to improve lordosing mobility of the lumbar spine and kyphosing mobility of the thoracic spine.
- Asymmetric exercises to improve postural corrections also in frontal and coronal plane and
- the physio-logic ADL posture.

The symmetric mobilizing exercises are performed repeatedly. These exercises can only be performed with the help of postural reflexes. Firstly lumbar lordosis is adopted actively and the pelvis is tilted forward while the upper trunk is slightly reclined backwards to improve thoracic kyphosis by reflex.

It is not the aim of the exercises to increase lumbar lordosis at the L5/S1 level as increased stress in this region would cause lower back pain. Therefore we aim at a lordosis at the L2 level enhancing the lordosis of the whole lumbar spine. We can ensure perfection of the exercise by ventralizing the lower ribs (Fig.14. on the right).

For the asymmetric postural correction in 3-D we can use exercises from the Schroth program modified to the principles of the physio-logic® exercise program (Fig.15a. middle).

Activities of daily living (ADL) are very important to change postural stereotype and for this reason the physio-logic ADL posture is trained in standing and walking (Fig. 15a. on the right). Therefore the patients are taught to perform the “Catwalk” which includes the basic principles of the physio-logic® program addressing the sagittal plane and the ADL

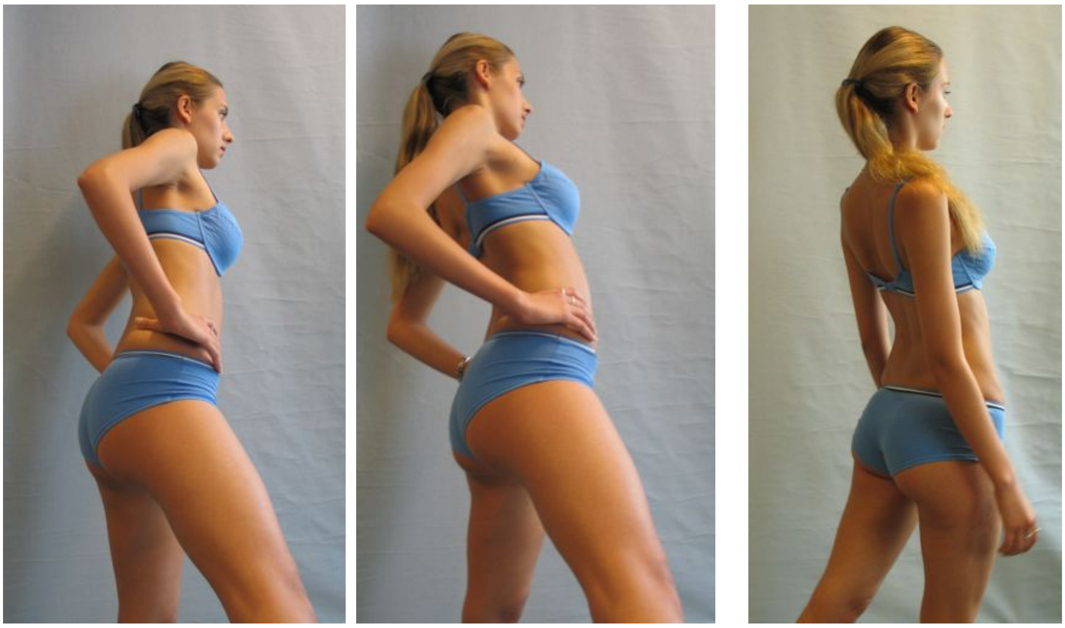

Fig. 15a. *Lateral aspect of the original Schroth exercise "Musclecylinder" on the left. Middle: "Musclecylinder physio-logic" and on the right the "Catwalk"; [modified from 116].*

(Activities of daily living) posture we call “Nuba” position. This position is derived from the normal upright standing and walking position the Nuba (natives of North Africa) perform regularly (Fig. 15b.).
There is no angle and range of thoracic kyphosis and lumbar lordosis actually defined when performing or maintaining the physio-logic® exercises. Muscle groups used for the exercises are not yet identified. This might be cause for further investigation.
The physio-logic® program can be used for the treatment of small scolioses as the only program (15 - 20°) and for the treatment of back pain as well, when the sagittal profile shows a malalignment.

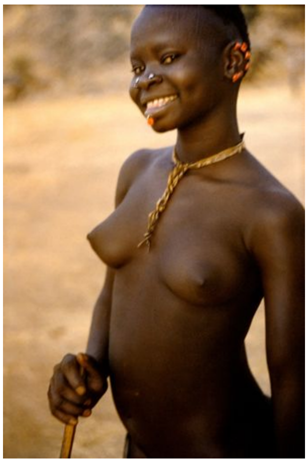

Fig. 15b. *Nuba girl as photographed by Leni Riefenstahl. (With kind permission by LRP)*

3D-Exercises made easy

The "3D-Exercises made easy" program is derived from the activities of daily living (ADL). These exercises can be performed in sitting and standing position. Basically a thoracic, a lumbar and a double major exercise can be performed addressing the different curve patterns in 3D. Of course there are also thorocolumbar curve patterns, but these can be adressed by taking the thoracic exercise (High thoracolumbar curve with apex TH 12; see also Fig. 16) or the lumbar exercise (Low thoracolumbar curve with apex L1).
The "3D-Exercises made easy" exercises have been shown easy to teach [117] and can be used for the treatment of small curves (15 - 30°) together with the physio-logic® program.

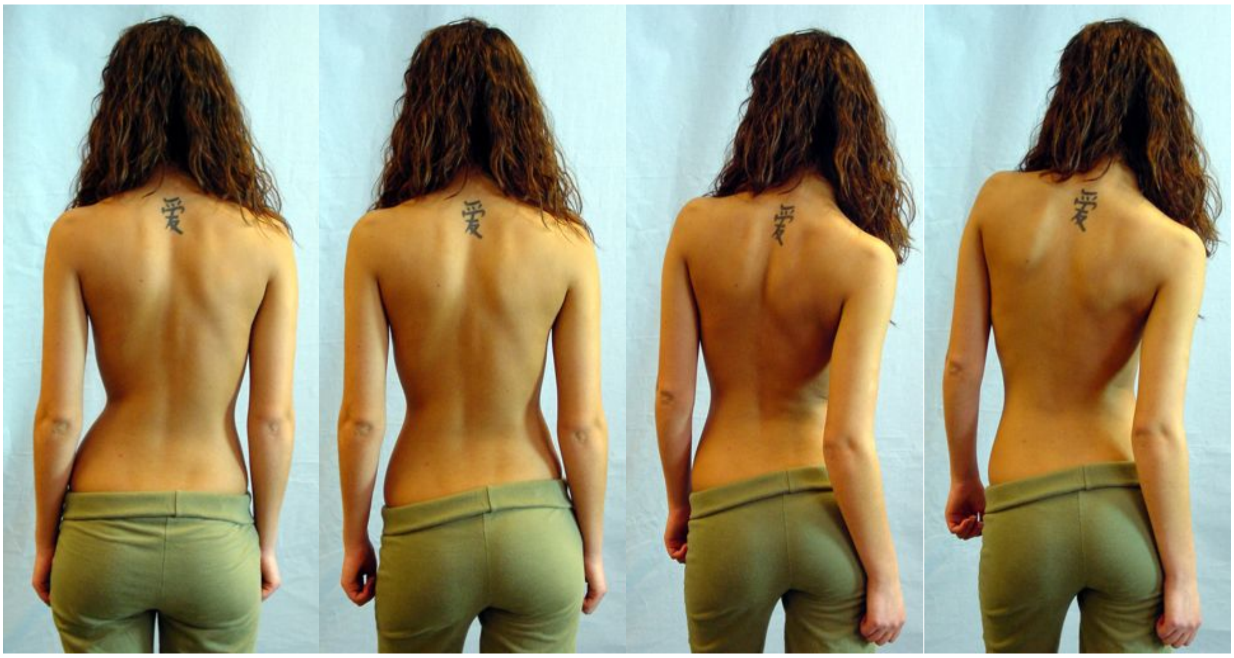

Fig. 16. *Patient with high thoracolumbar curve treated like thoracic, according to the concept "3D-exercises made easy":*
(1) Pelvic overcorrection, (2) Shoulder retraction with simultaneous alignment of the sagittal profile, (3) Breathing and (4) Stabilisation via trunk muscle tension in the corrected position.

The Schroth program

The exercise program according to Schroth is described at length in several publications [88,107] and the idea of in-patient rehabilitation has been subject of many scientific investigations [88]. The advantage of this program is the specificity of postural corrections designed for different curve patterns and the introduction of effective mechanisms to increase 3-D postural correction in scoliosis [1-3].
Basically the aim of physiotherapy is to enable the patient to attain postural corrections for him or herself, with the exclusive use of trunk muscles, although dynamic passive forces (manual help by the therapist) and static forces can be used.
When the patient has gone through the principles of physio-logic, pattern specific ADL correction and 3D-made-easy, the acqisition of the pattern specific Exercises from the "New Power Schroth" program are very easy. The patient is also taught how to make corrections or adjustments to his or her posture in different parts of the body by means of exteroceptive stimulation or by provoking a reaction of balance (Fig. 17 and 18.). Equally important are the proprioceptive stimulations, both through manual passive corrections on the deformed trunk and through changes in articular position, passive traction-compression movements or manual relaxation, activation or elongation tests of elastic structures.

Schroth classically considered 5 principles for correction:

Axial elongation
Deflexion
Derotation
Facilitation
Stabilisation

Active axial elongation
Active axial elongation is achieved by self-elongation. The patient has to try and elongate as much as possible by the active strength of the trunk muscles.
As we know today that axial elongation leads to a flatback deformity, this kind of longitudinal correction no longer plays a part in the "New Power Schroth" program. The restoration of a physiological sagittal profile in patients with AIS plays a most important role, while in patients with kyphoscoliosis active axial elongation still is performed.
In kyphoscoliosis patients this can also be reinforced by using the old Schroth exercises while hanging on the wallbar. These,however are not used in patients with AIS anymore!

Deflexion and Derotation

These terms are used no more within the treatment of AIS patients using the "New Power Schroth" program. Today we are not focussing on single curves, but on effective corrective movements mainly using the shift of the shoulder girdle against the pelvic girdle. We do no more provide special exercises for 3- or 4-curve patterns, but we adapt the individual exercises to the curve pattern of the patient by applying pelvic corrections accordingly. The whole spine is important and not he single curve and therefore the classical complex principles of the original Schroth program seem to be outdated today.

Schroth's rotatory breathing

During physiological respiratory movement, all regions of the trunk, thorax and abdomen expand, with the purpose of increasing the volume and the air intake to the lungs. The thoracic cage and the inhalation muscles on one hand and the lung mass on the other, form two elastic systems in opposite directions, joined by two sheets and a pleural space. The scoliosis deformation process causes morphological changes in the trunk. Some areas of the trunk protrude or become convex, and others sink in or become concave. Breathing mechanics do not function normally. The deformity causes an imbalance in all muscles of the trunk. This imbalance has been established in second place in the deformation process and is both a morphological imbalance (the origin and insertion of certain muscular groups move apart and therefore stretch, while the origin and insertion of other muscles move together, resulting in a shortening) and also a functional imbalance (the streched muscles are subject to sustained tension showing a higher EMG activity, shortened muscles are not).

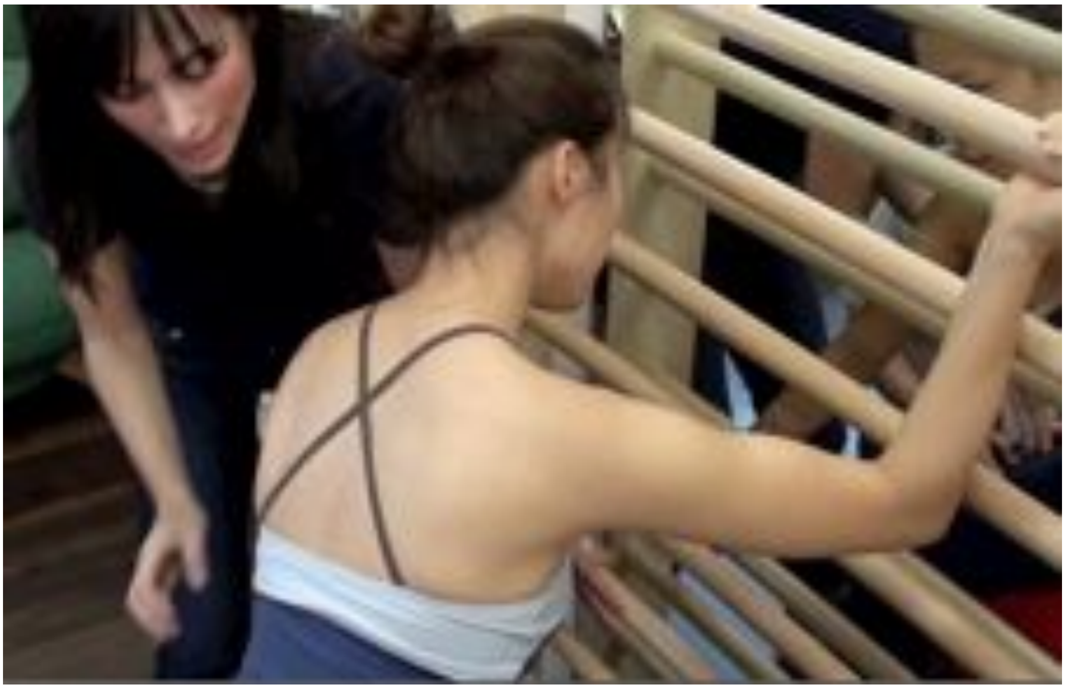

Fig. 17. *Ventral pressure against the sternum by the therapist enforces the rekyphosation of the thoracic flatback during the "New Power Schroth" exercise on the wallbar (50 times exercise).*

The result of this morphological and functional muscular imbalance, secondary to scoliosis, is the cause of modified respiratory mechanics, which in turn, influences the evolutional process of the deformity, as asymmetric muscular forces are produced which increase the deforming forces. Whether the muscular imbalance is primary or secondary, once the disorder has been established and this becomes progressive, a vicious circle is set up in which the deformity increases the imbalance and the imbalance increases the deformity.
The passively increased tone of the overstretched muscles stops the exhalation retraction of the convex areas, causing what is called an inhalation blockage in these areas. On the other hand, in concave areas, the elastic structures adapt to a situation of retraction, with a mechanical advantage over inhalation muscles, which explains the greater exhalation retraction of these areas. During inhalation, all areas of the trunk expand to a greater or lesser degree, and during exhalation, the convex areas remain blocked in inhalation and the concave areas retract again to a situation of greater exhalation. This phenomenon results in increase of the deformity in exhalation, compared to inhalation which reduces it.
Apart from passive and active corrections and asymmetric output positions for exercises, the therapeutic effect on muscular balance is achieved by maintaining the corrections during the exhalation phase.
The patient during Schroth exercises has to tense the convexities and to guide his breath to the concavities of the trunk. The effect of this work by the patient is a proprioceptive reinforcement of the sensation of trunk detorsion over the vertebral column. This technique is called Schroth's rotatory breathing. Stimulation by the therapist is very important. With a slight pressure of the fingers, the therapist's hand slides over the sunken areas and moves in a corrective direction still in the "New Power Schroth" program.

Facilitation

The neurophysiological bases of facilitation are not included in this chapter. They have already been considered and described elsewhere [109]. What is interesting to consider at this point is their practical aspect, meaning where the proprio and exteroceptive stimuli are applied to support postural correction. Within the "New Power Schroth" program we use this technique of stimulation rarely so as to allow the patient to experience the movements more by himself (Experiencial Learning). The stimuli are only used when an exercise is taught for the first time.

Stabilisation

Stabilisation refers to the maintenance of the correction producing a theoretically isometric tension in the exhalation phase. It is already explained in the section on respiratory mechanics, how muscular tension produced during exhalation with the maintenance of the correction has an active elongation effect on shortened muscles and an activation in a

shorter position on the over-stretched musculature. In this way, the length and muscular sufficiency are recuperated. To generate greater isometric muscular tension, the patient can use different tools and resistance by the therapist as well. Today we try to make it simple. Like in the 3D-made-easy exercises the patient is asked to perform a trunk muscle tension in the "New Power Schroth" program. This is done after the best possible postural correction is achieved in the exhalation phase of the exercises.

General Principle of 3D Correction of the Column

The general correction principle of scoliosis in the "New Power Schroth" program could be stated as "shifting the shoulder girdle against the pelvic girdle". The shift is performed before or after the pelvis has been corrected according to the individual curve pattern the patient has.

During this frontal plane correction the patient is taught how to maintain the sagittal correction, maintain a lordosis in the lumbar and kyphosis in the thoracic part of the trunk (see Fig. 17.) also with the help of exteroceptive stimuli.

Additionally the breath of the patient is guided into the concave areas of the trunk during inspiration. also this at first is supported by certain tactile aids given by the therapist.

The steps of correction within the "New Power Schroth" program are very simple and easy to learn after the first three parts of the program have been acquired, namely the (1) physio-logic® program, the (2) correction of ADL and (3) 3D-made-easy.

These steps are:

1. Acquisition of the starting position.
2. Shift of the shoulder girdle against the pelvic girdle.
3. Pattern specific pelvic correction
4. Sagittal correction
5. Rotational breathing
6. Stabilisation

It is most important not to lose the postural correction gained during the exercise (after having run through steps 1- 6) at the end, but to keep it and improve it and repeat point 2 - 6 several times trying always to improve the previous postural correction achieved.

Within the "New Power Schroth" program exercise program pattern-specific correction mechanisms are taught depending on the clinical findings of the patient. Although the number of possible curve patterns seems quite high, in the Schroth system we consider two basic curve patterns, which in practice seems enough to address most of the typical findings a scoliosis can present with [107]:

1. *Functional 3 curve pattern* (Fig. 19. and 20.) and the
2. *Functional 4 curve pattern* (Fig. 21.)

However, according to the augmented classification demonstrated in Fig. 9. there is a more detailed way to look at the two principal curve patterns named above:

1. *Functional 3 curve pattern with decompensation*
2. *Functional 3 curve pattern with decompensation (high thoracolumbar T 12)*
3. *Functional 3 curve pattern with neutral pelvis*
4. *Functional 3 curve pattern with long lumbar counter curve (treated like 4 curve)*
5. *Functional 4 curve pattern (double major)*
6. *Functional 4 curve pattern (single lumbar)*
7. *Functional 4 curve pattern (single thoracolumbar L 1)*

The Schroth exercise program has proven to be relatively effective especially in the environment of in-patient rehabilitation [6,9.88,114,115], however long-term effects of such treatment are not yet demonstrated. It is also important to state, that the old studies relying on a pre- / post design were performed in the 80's or early 90's of the last century. At that time the bracing standard was very low when compared to today's correction effects. Therefore for the patients during growth an intensive treatment with exercises seemed very important. On the other hand the patients in the 70's or 80's were more persistent and therefore were able to exercise longer than today's population of adolescents with scoliosis.
Today the bracing standard is constantly improving with failure rates of less than 5%. Therefore physiotherapy during the growth spurt can regarded as being secondary.

The three most important aids to improve postural perception

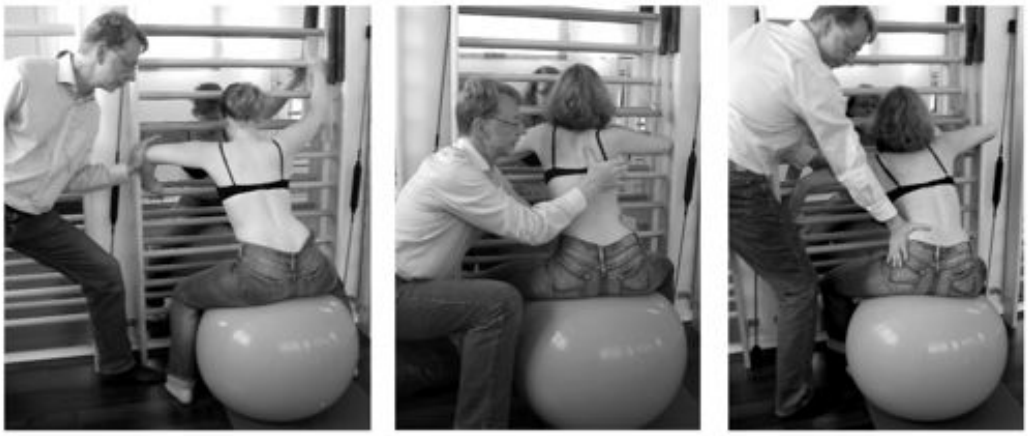

Fig. 18. *Tactile aids to improve postural perception and the execution of the exercises.*
(1) resistance against the corrective shift.
(2) resistance against the spiral shoulder girdle correction and
(3) resistance against 4 curve pelvic correction.

3B

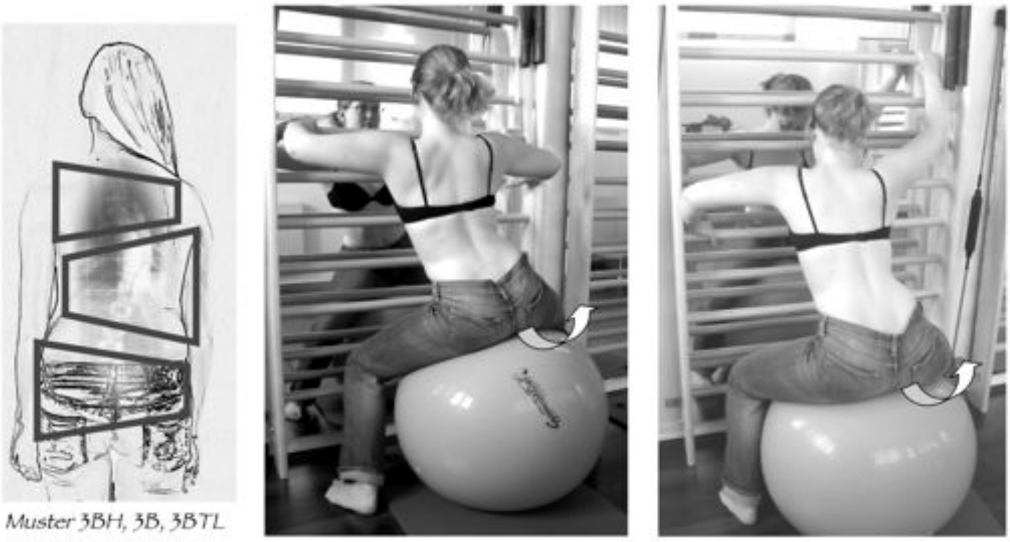

Muster 3BH, 3B, 3BTL

Fig. 19. Example of an exercise for a functional 3 curve scoliosis with decompensation (3B). For recompensation the pelvic shift is crucial. On the left the typical appearance of a 3 curve scoliosis with decompensation, in the middle execution for kyphoscoliosis and on the right for AIS.

3B

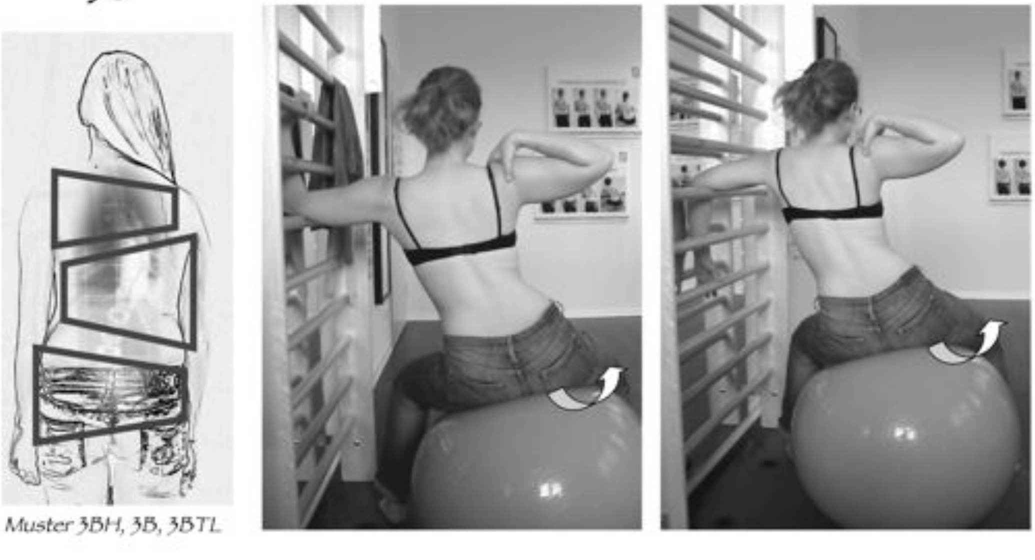

Muster 3BH, 3B, 3BTL

Fig. 20. Example of an exercise for a functional 3 curve scoliosis with decompensation (3B). For recompensation the pelvic shift is crucial. On the left the typical appearance of a 3 curve scoliosis with decompensation, in the middle execution for kyphoscoliosis and on the right for AIS with flatback.

4B

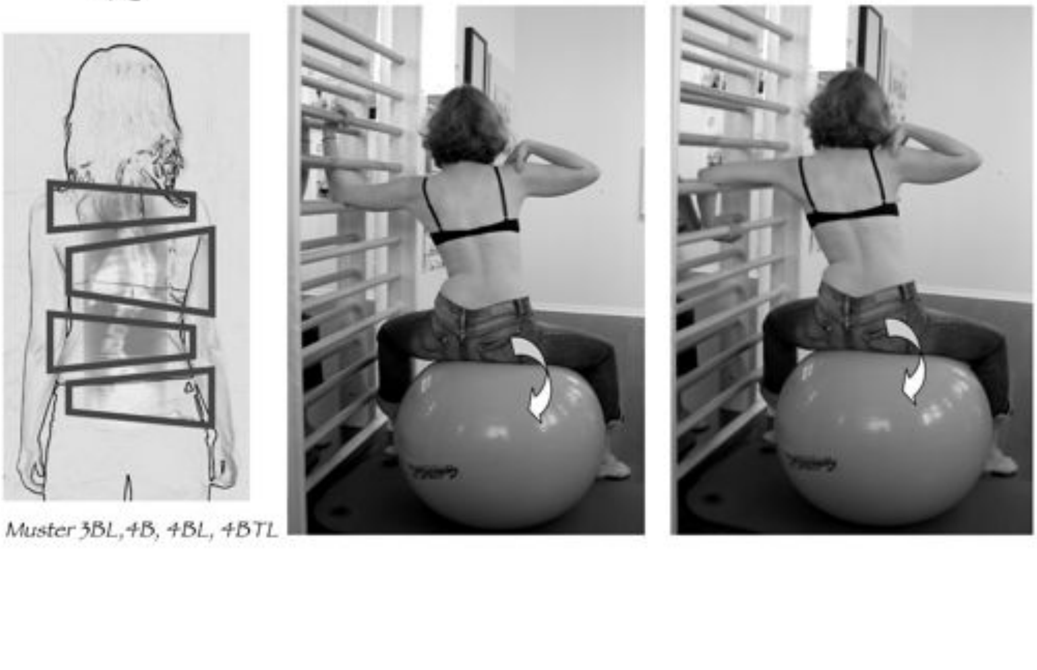

Muster 3BL, 4B, 4BL, 4BTL

Fig. 21. Example of an exercise for a functional 4 curve scoliosis without decompensation (4B). Trunk shift and the pelvic correction are the main principles in frontal plane. On the left the typical appearance of a 4 curve scoliosis without decompensation, in the middle execution for kyphoscoliosis and on the right for AIS with flatback.

Due to our improved understanding of the scoliotic influence on the sagittal profile [96,97,98,101,116] a combination of the Schroth and the physio-logic® principles seem desirable in patients with Cobb angles of > 30°. So in future more emphasis should be given to sagittal corrections. As uncontrolled autoelongation may lead to flatback, we should focus on the possibilities of 3-D postural corrections without elongation.
If we regard a combination of "physio-logic®", "3D-exercises made easy", and the "New Power Schroth" program as "Best Practice" we will have to compare the described treatment strategies to other methodological approaches:

Methode Lyonaise / SEAS
Dobosiewicz
Side Shift

The Lyonaise Method aims at 3D postural correction and an improvement of skills the patient needs for autocorrection. The postural correction however is not comparable to that achieved by use of the Schroth technique (Fig. 12.). The sagittal correction is mainly corrected with the help of certain exercises in the brace. Without the lordosing counteraction of the lumbar pad many exercises from the Lyonaise school lead to a total kyphosis which cannot be regarded as effective [10,112].
The Dobosiewicz Method is not well described in international literature. In Germany little is known about this method of physiotherapy. One aim of this method is the rekyphosation of thoracic flatback. During the exercises the patients are forced into a forward bending position leading to a kyphosation of the whole spine [118]. How the relordosation of the lumbar profile is established is still an open question for this method of treatment. The only scientific investigation published so far seems questionable, when the average patient from the sample presented would not be treated at all according to our guidelines [118].
The Side Shift technique addresses the deformity in the frontal plane only. Meanwhile we have gained evidence that the postural correction can be improved when lumbar lordosis as well as thoracic kyphosis is restored [101,116]. The frontal deviation can be regarded as the secondary deformity [96,97,98] and for this reason Side Shift exercises have to be regarded as the second choice when performed as the sole form of exercise.
New exercise programs presented by Negrini et al. [119] and Romano et al. [120] are derived from the Lyonaise school. In a personal communication, Negrini (2004) reported concerns against mobilisation, however the so called SEAS 02 exercises, allegedly developed 2002, in the year 2006 contain mobilisation techniques [119].
Up to now there are no prospective outcome studies showing the SEAS program to reduce the progression risk or improve other signs and symptoms of scoliosis. Therefore comparing two programs without evidence in a randomized controlled study does not

seem to make sense [119]. Furthermore when the ADL-exercises described need months to be aquired by the patient [120] it is the question as to whether it is worthwile at all to teach them.

According to latest knowledge, exercise programs should be easy to understand, simple and effective. Mobilisation techniques are necessary to change loads on the vertebra.

Among many of the recent publications on physiotherapy many clinical studies are of questionable treatment indications and therefore cannot contribute to the body of evidence already existent [121]. So evidence for physiotherapy is still in question.

References

1. FUSCO C, ZAINA F, ATANASIO S, ROMANO M, NEGRINI A, NEGRINI S: Physical exercises in the treatment of adolescent idiopathic scoliosis: an updated systematic review. *Physiother Theory Pract.* 2011; Jan; 27(1):80-114.

2. WEINSTEIN SL: Natural history. *Spine,* 24: 2592-2600, 1999.

3. FERRARO C, MASIERO S, VENTURIN A *et al.*: Effect of exercise therapy on mild idiopathic scoliosis. Preliminary result. *Europa Medico Physica*, 34: 25-31, 1998.

4. RIGO M, QUERA-SALVA G, PUIGDEVALL N: Effect of the exclusive employment of physiotherapy in patients with idiopathic scoliosis. In: *Proceedings Book III of the 11th International Congress of the World Confederation for Physical Therapy* (London), pp 1319-1321, 1991.

5. WEISS HR: Influence of an in-patient exercise program on scoliotic curve. *Italian Journal of Orthopedics and Traumatology*, 18: 395-406, 1992.

6. WEISS HR, LOHSCHMIDT K, EL-OBEIDI N *et al.*: Preliminary results and worst-case analysis of in-patient scoliosis rehabilitation. *Pediatric Rehabilitation*, 1: 35-40, 1997.

7. STONE B, BEEKMAN C, HALL V *et al.*: The effect of an exercise program on change in curve in adolescents with minimal idiopathic scoliosis. A preliminary study. *Physical therapy*, 59: 759-763, 1979.

8. DEN BOER WA, ANDERSON PG, LIMBEEK J *et al.*: Treatment of idiopathic scoliosis with side-shift therapy: an initial comparison with a brace treatment historical cohort. *European Spine Journal*, 8: 406-410, 1999.

9. WEISS HR, WEISS G and PETERMANN F: Incidence of curvature progression in idiopathic scoliosis patients treated with scoliosis in-patient rehabilitation (SIR): an age- and sex-matched controlled study. *Pediatric Rehabilitation*, 6: 23-30, 2003.

10. DUCONGE P: La rééducation de la scoliose. Mythe ou réalité? *Résonnances Européennes du Rachis*, 10 : 1229-1236, 2002.

11. KLISIC P and NIKOLIC Z: Scoliotic attitudes and idiopathic scoliosis. In: *Proceedings of the International Congress on Prevention of Scoliosis in Schoolchildren* (Milan: Edizioni Pro Juventute), pp 91-92, 1985.

12. MOLLON M and RODOT JC: Scolioses structurales mineures et kinésithérapie. Etude statistique comparative des résultats. *Kinésithérapie Scientifique*, 244: 47-56, 1986.

13. REILLY RP and FINDLEY TW: Research in physical medicine and rehabilitation. IV. Some practical designs in applied research. *American Journal of Physical Medicine and Rehabilitation*, 70 (suppl. 1): 31-36, 1991.

14. FOCARILE FA, BONALDI A, GIAROLO M *et al.*: Effectiveness of nonsurgical treatment for IS; overview of available evidence. *Spine*, 16: 395-401, 1991.

15. LANTZ CA: Conservative management of scoliosis. *Chiropractic*, 9: 100-107, 1995.

16. ROWE D, BERNSTEIN S, RIDDICK MF, ADLER F, EMANS JB and GARDNER-BONNEAU D: A meta-analysis of the efficacy of nonoperative treatment for IS. *Journal of Bone and Joint Surgery [Am.]*, 79: 664-669, 1997.

17. WOOLF S: Screening for adolescent idiopathic scoliosis. *Journal of the American Medical Association*, 269: 2667-2672, 1993.

18. LONSTEIN JE and CARLSON JM: The prediction of curve progression in untreated idiopathic scoliosis during growth. *Journal of Bone and Joint Surgery [Am.]*, 66: 1061, 1994.

19. BROOKS HL, AZEN SP, GERBERG E, BROOKS R and CHAN L: Scoliosis: a prospective epidemiological study. *Journal of Bone and Joint Surgery [Am.]*, 57: 968-972, 1975.

20. ASCANI E, BARTOLOZZI P and LOGROSCINO CA *et al.*: Natural history of untreated IS after skeletal maturity. *Spine*, 11: 784-789, 1986.

21. BJURE J and NACHEMSON A: Non-treated scoliosis. *Clinical Orthopedics Related Research*, 93: 44-52, 1973.

22. BJERKREIM R and HASSAN I: Progression in untreated IS after the end of growth. *Acta Orthopedica Scadinavia*, 53: 897-900, 1982.

23. BUNNELL WP: The natural histiory of IS before skeletal maturity. *Spine*, 11: 773-776, 1986.

24. COLLIS DK and PONSETI IV: Long-term follow-up of patients with IS scoliosis non treated surgically. *Journal of Bone and Joint Surgery [Am.]*, 51-A: 425-445, 1969.

25. FOWLES JV, DRUMMOND DS, L'ECUYER S *et al.*: Untreated scoliosis in the adult. *Clinical Orthopedic Related Research*, 134: 212-224, 1978.

26. NACHEMSON A: A long-term follow-up study of nontreated scoliosis. *Acta Orthopedica Scandinavia*, 39: 446-476, 1968.

27. PONSETI IV and FRIEDMAN B: Prognosis in IS. *Journal of Bone and Joint Surgery [Am.]*, 32: 381-395, 1950.

28. HAWES M: The use of exercise in the treatment of scoliosis: an evidence-based critial review of the literature. *Pediatric Rehabilitation,* 6*:* 171-182, 2003.

29. KEIM MA: The Adolescent Spine, 2nd edn (New York, Heidelberg, Berlin: Springer-Verlag), 1987.

30. JAREMKO JL, PONCET P, RONSKY J *et al.*: Estimation of spinal deformity in scoliosis from torso surface cross sections. *Spine* 26: 1583-1591, 2001.

31. KOJIMA T and KUROKAWA T: Quantification of three-dimensional deformity of IS. *Spine*, 17: 22-29, 1992.

32. PONCET P, DANSERAU J and LABELLE H: Geometric torsion in IS; three-dimensional analysis and proposal for a new classification. *Spine* 26: 2235-2243, 2001.

33. VILLEMURE I, AUBIN CE, GRIMARD G *et al*: Progression of vertebral and spinal three-dimensional deformities in AIS. A longitudinal stuy. *Spine* 26: 2244-2250, 2001.

34. ROAF R: Scoliosis (Baltimore, MD.: Williams and Wilkins), 1966.

35. STOKES IAF: Hueter-Volkmann Effect; state of the art reviews, *Spine* 14: 349-357, 2000.

36. STOKES IAF and GARDNER-MORSE M: The role of muscles and effects of load on growth. *Research into Spinal Deformity*, 4: 314-317, 2002.

37. KOROVESSIS P, PIPEROS, G, SIDIROPOULOS P *et al*: Adult idiopathic lumbar scoliosis : a formula for prediction of progression and review of the literature. *Spine* 19: 1926-1932, 1994.

38. WEINSTEIN SL and PONSETI IV: Curve progression in IS. *Journal of Bone and Joint Surgery*, 65-A: 447-455, 1983.

39. WEINSTEIN SL, DOLAN LA, SPRATT KF *et al*: Health and function of patients with untreated IS: a 50-year natural history study. *JAMA*, 289: 559-567, 2003.

40. BROWN D: The pain drawing in AIS. *Proceedings of the Scoliosis Research Society, 36th Annual Meeting*, Cleveland, OH, 2001.

41. BALAGUE, F., DUTOIT, G. and WALDBURGER, M.: Low back pain in schoolchildren. An epidemiological study. *Scandinavian Journal of Rehabilitation Medicine* 20: 175-179, 1988.

42. EDGAR, M.A.: Back pain assessment from a long term follow-up of operated and unoperated patietns with AIS. *Spine* 4: 519-521, 1979.

43. FAIRBANK, J.C., PYSENT, P.B., VAN POORTVLIET, J. A. *et al*.: Influence of anthropometric factors and joint laxity in the incidence of adolescent back pain. *Spine* 9: 461-464, 1984.

44. KOSTUIK, J.P., BENTIVOGLIO, J.: The incidence of low-back pain in adult scoliosis. *Spine*, 6: 268-273, 1981.

45. JACKSON, R.P., SIMMONS, E.H. and STRIPINIS, D.: Incidence and severity of back pain in adult idiopathic scoliosis. *Spine* 8: 749-756, 1983.

46. MAYO, N.E., GOLDBERG, M.S., POITRAS, B. et al.: The Ste-Justine AIS cohort study. Part III: Back pain. *Spine* 14: 1573-1581, 1994.

47. NATASI, A.J., LEVINE, D.B. and VELISKAKIS, K.P.: Pain patterns in AIS. *Journal of Bone and Joint Surgery* 54-A: 1575, 1972

48. NILSONNE, U., LUNDGREN, K.D.: Long term prognosis in IS. *Acta Orthopedica Scandinavica* 39: 456-465, 1968.

49. RAMIREZ, N., JOHNSTON, C.E. and BROWNE, R.H.: The prevalence of back pain in children who have IS. *Journal of Bone and Joint Surgery* 79-A: 364-368, 1997.

50. VITALE, M.G., LEVY, D.E., JOHNSON, M.G. et al.: Assessment of quality of life in adolescent patients with orthopedic problems: are adult measures appropriate? *Journal of Pediatric Orthopedics*, 21: 622-628, 2001.

51. WEINSTEIN, S.L., ZAVALA, D.C. and PONSETI, I.V.: Idiopathic scoliosis long-term follow-up and prognosis in untreated patients. *Journal of Bone and Joint Surgery* 63-A: 702-712, 1981.

52. RINSKY, L.A. and GAMBLE, J.G.: AIS. *Western Journal of Medicine* 148: 182-191, 1988.

53. GOLDBERG, M.S., MAYO, N.E., POITRAS, B. et al.: The Ste. Justine AIS cohort study: II. Perception of health, self and body image, and participation in physical activities. *Spine* 19: 1562, 1994.

54. WEINSTEIN, S.L.: Long term follow-up of pediatric orthopedic conditions; natural history and outcomes of treatment. *Journal of Bone and Joint Surgery* 82-A: 980-990, 2000.

55. BENGSTON, G., FALLSTROM, K., JANSSON, B. *et al*.: A psychological and psychiatric investigation of the ajustment of female scoliosis patients. *Acta Psychiatrica Scandinavica* 50: 50-59, 1974.

56. CLAYSON, D. and LEVINE, D.B.: AIS: personality patterns and effects of corrective surgery. *Clinical Orthopedic Related Research* 116: 99-102, 1976.

57. CLAYSON, D., LUZ-ALTERMAN, S., CATALETTO, M.M., *et al*.: Long-term psychological sequelae of surgically vs. nonsurgically treated scoliosis. *Spine* 12: 983-987, 1987.

58. EDGAR, M.A. and METHA, M.H.: Long term follow-up of fused and unfused IS. *Journal of Bone and Joint Surgery* 70-B: 712-716, 1986.

59. FALLSTROM, K., COCHRAN, T. and NACHEMSON, A.: Long term effects on personality development in patients with AIS; influence of type of treatment. *Spine* 11: 756-758, 1986.

60. HECKMAN, L.A., SCHATZINGER, L.A., NASH, C.L. *et al*.: Emotional adjustment in scoliosis. *Clinical Orthopedic Related Research* 125: 145-150, 1977.

61. KAHANOVITZ, N. and WEISER S.: The psychological impact of IS on the adolescent female; a preliminary multi center study. *Spine* 14: 483-487, 1989.

62. MACLEAN, W.E., GREEN, N.E., PIERRE, C.B. *et al.*: Stress and coping with scoliosis: psychological effects on adolescents and their families. *Journal of Pediatric Orthopedics* 9: 257-260, 1989.

63. PAYNE WK, OGLIVIE JW, RESNICK MD *et al*: Does scoliosis have a psychological impact and does gender make a difference? *Spine* 22: 1380-1384, 1997.

64. BOWEN RM: Respiratory management in scoliosis. In: J. Lonstein, D. Bradford, R. Winter *et al*. (editors) Moe's Textbook of Scoliosis and Other Spinal Deformities, 3rd edn (Philadephia: WB Saunders), pp 572-581, 1995.

65. GEORGE RB, LIGHT RW, MATTHAY MA et al: Chest Medicine: Essentials of Pulmonary and Critical Care Medicine, 3rd edn (Baltimore: Williams and Wilkins), 1995.

66. SEATON A, SEATON D and LEITCH AG: *Crofton* and Douglas's Respiratory Diseases, 5th edn (Oxford: Blackwell Science), p 90, 2000.

67. FRASER RS, MÜLLER NL, COLMAN N et al: Fraser and Pare's Diagnosis of Diseases of the Chest, 4th edn (Philadelphia: W.B. Saunders Company), 1990.

68. HITOSUGI M, SHIGETA A and TAKATSU A: An autopsy case of sudden death in a patient with IS. *Medicine Science and the Law* 40: 175-178, 2000.

69. MURRAY JF and NADEL JA: Textbook of Respiratory Medicine, 3rd edt. (Philadelphia: W.B. Saunders), 2000.

70. PEHRSSON K, LARSSON S, ODEN A *et al*: Long term follow-up of patients with untreated scoliosis. A study of mortality causes of death and symptoms. *Spine* 17: 1091-1096, 1992.

71. SCHNEERSON JM, SUTTON GC and ZORAB PA: Causes of death, right ventricular hypertrophy, and congenital heart disease in scoliosis. *Clinical Orthpedic Related Research* 135: 52-57, 1978.

72. BOYER J, AMIN N, TADDONIO R *et al*: Evidence of airway obstruction in children with IS. *Chest* 109: 1532-1535, 1996.

73. CHONG KC, LETTS RM and CUMMING GR: Influence of spinal curvature on exercise capacity. *Journal of Pediatric Orthopedics* 1: 251-254, 1981.

74. DIROCCO P, BREED AL, CARLIN JE et al: Physical work capacity in adolescents with mild IS. *Archives of Physical Medicine Rehabilitation* 64: 476-479, 1983.

75. DIROCCO P and VACCARO P: Cardiopulmonary functioning in adolescent patients with mild IS. *Archives of Physical Medicine Rehabilitation* 69: 198-199, 1988.

76. HAWES MC and BROOKS WJ: Improved chest expansion in IS after intensive, multiple modality, nonsurgical treatment in adult. *Chest* 120: 672-674, 2001.

77. KEARON C, VIVIANI GR and KILLIAN KJ: Factors influencing work capacity in thoracic AIS. *American Review in Respiratory Diseases* 148: 295-303, 1993.

78. KESTEN S, GARFINKEL SK, WRIGHT T *et al*: Impaired exercise capacity in adults with moderate scoliosis. *Chest* 99: 663-666, 1991.

79. OGLIATI R, LEVINE D, SMITH JP et al: Diffusing capacity in IS and its interpretation regarding alveolar development. *American Reviews in Respiratory Disease* 126: 229-234, 1982.

80. SMYTH RJ, CHAPMAN KR, WRIGHT TA *et al*: Pulmonary function in adolescents with mild IS. *Thorax* 39: 901-904, 1984.

81. SMYTH RJ, CHAPMAN KR, WRIGHT TA *et al*: Ventilatory patterns during hypoxia, hypercapnia, and exercise in adolescents with mild scoliosis. *Pediatrics* 77: 692-696, 1986.

82. SZEINBERG A, CANNY GJ, RASHED N *et al*: Forced VC and maximal respiratory pressures in patients with mild and moderate scoliosis. *Pediatric Pulmonaries* 4: 8-12, 1988.

83. WEBER B, SMITH JP, BRISCOE WA *et al*: Pulmonary function in asymptomatic adolescents with IS. *American Reviews in Respiratory Disease* 111: 389-397, 1975.

84. BRANHWAITE MA: Cardiorespiratory consequences of unfused IS. *British Journal of Diseases of the Chest*, 80:360-369, 1986.

85. DAVIES G and REID L: Effect of scoliosis on growth of alveoli and pulmonary arteries and on the right ventricle. *Archives of Disease in Childhood* 46: 623-632, 1971.

86. DRUMMOND DS: Spinal deformity: natural history and the role of screening. In: D.S. Bradford and R.M. Densinger (editors) *The Pediatric Spine* (Stuttgart, NY: Georg Thieme Verlag), pp 167-180, 1985.

87. LEATHERMAN K and DICKSON R: *The Management of Spinal Deformities* (London, Boston, Singapore, Sydney, Toronto, Wellington:Wright Press), 1988.

88. WEISS HR: Skolioserehabilitation, Qualitätssicherung und Patientenmanagement. (Stuttgart: Thieme), 2000.

89. WEISS HR: The effect of an exercise program on VC and rib mobility in patients with IS. *Spine*, 16: 88-93, 1991.

90. WEISS HR, BICKERT W: Veränderungen elektrokardiographisch objektivierbarer Parameter der Rechtsherzbelastung erwachsener Skoliosepatienten durch das stationäre Rehabilitationsprogramm nach Schroth. *Orthopädische Praxis*, 32:450-453, 1996.

91. WEISS HR, NEGRINI S, RIGO M, KOTWICKI T, GRIVAS TB, MARUYAMA T and members of the SOSORT: SOSORT Consensus Paper Part I: Physical Exercises in the Treatment of Idiopathic Scoliosis. Discussed at the SOSORT consensus meeting, Milano, January 2005.

92. WEISS HR: Imbalance of electromyographic activity and physical rehabilitation of patients with idiopathic scoliosis. *European Spine Journal* 1: 240-243, 1993.

93. DANIELSSON AJ, WIKLUND I, PEHRSSON K, NACHEMSON AL: Health-related quality of life in patients with adolescent idiopathic scoliosis: A matched follow-up at least 20 years after treatment with brace or surgery. *European Spine Journal*, 10: 278-288, 2001.

94. WEISS HR: La rehabilitatión de la escoliosis. Paidotribo, Barcelona, 2003.

95. STOKES IAF: *Die Biomechanik des Rumpfes*. In: H.R. Weiß: Wirbelsäulendeformitäten. Konservatives Management. München, Pflaum, pp 49-77, 2003.

96. DEACON P, FLOOD BM and DICKSON RA: Idiopathic scoliosis in three dimensions: a radiographic and morphometric analysis. *Journal of Bone and Joint Surgery* 66-B: 509-512, 1984.

97. TOMASCHEWSKI R: Die funktionelle Behandlung der beginnenden idiopathischen Skoliose. Habilitation thesis, Halle, 1987.

98. WEISS HR and LAUF R: Impairment of forward flexion – physiological or the precursor of spinal deformity? In: M. D'Amico, A. Merolli and G.C. Santambrogio (editors) Three Dimensional Analysis of Spinal Deformities (Amsterdam: IOS Press), pp 307-312, 1995.

99. BURWELL RG: Aetiology of idiopathic scoliosis: current concepts. *Pediatric Rehabilitation* 6: 137- 170, 2003.

100. RASO VJ: Biomechanical factors in the etiology of idiopathic scoliosis. *Spine: State of the Art Reviews* 14: 335-338, 2000.

101. WEISS HR, DALLMAYER R, GALLO D: Sagittal Counter Forces (SCF) in the treatment of Idiopathic Scoliosis – A Preliminary Report. *Pediatric Rehabilitation* 9: 24-30, 2006.

102. RIGO M, WEISS HR: Korsettversorgungsstrategien in der Skoliosebehandlung. In: H.R. Weiss Wirbelsäulendeformitäten, Konservatives Management (München, Pflaum), pp 251-287, 2003.

103. KLAPP R: Funktionelle Behandlung der Skoliose. Jena: Fischer; 1907.

104. NIEDERHÖFFER L: Die Behandlung von Rückgratverkrümmungen (Skoliose) nach dem System Niederhöffer. Berlin, Osterwieck, 1942.

105. OZARCUK L: Grundlagen der Skoliosebehandlung mit der Propriozeptiven Neuromuskulären Fazilitation. In: WEISS HR: Wirbelsäulendeformitäten (Vol. 3), Gustav Fischer Verlag, Stuttgart, pp 11-30, 1994

106. WEISS HR: Peripher Evozierte Haltungsreaktionen – Erste Erfahrungen. In: WEISS HR: Wirbelsäulendeformitäten (Vol. 3), Gustav Fischer Verlag, Stuttgart, pp 53-58, 1994.

107 LEHNERT-SCHROTH CH: Dreidimensionale Skoliosebehandlung. 7th. edition. Stuttgart: Urban & Fischer, 2007.

108. TOMASCHEWSKI R: Die Frühbehandlung der beginnenden idiopathischen Skoliose In: WEISS HR: Wirbelsäulendeformitäten (Vol. 2), Gustav Fischer Verlag, Stuttgart, pp 51-58, 1992

109. RIGO M, QUERA-SALVA G, PUIGDEVALL N: Auswirkungen der Schrothschen Dreh-Winkel Atmung auf die dreidimensionale Verformung bei idiopathische Thorakalskoliose. In: WEISS HR: Wirbelsäulendeformitäten (Vol. 3), Gustav Fischer Verlag, Stuttgart, pp 87-92, 1994.

110. WEISS HR, RIGO M: Befundgerechte Physiotherapie bei Skoliose. Pflaum, München, 2001.

111. NEGRINI A: Forschungsdaten und ihre Konsequenzen für die Krankengymnastik. In: WEISS HR: Wirbelsäulendeformitäten (Vol. 2), Gustav Fischer Verlag, Stuttgart, pp 85-88, 1992.

112. DUCONGÉ P: Der Skoliotische Flachrücken. Krankengymnastische Therapieansätze. In: WEISS, H.R.: Wirbelsäulendeformitäten (Vol. 2), Gustav Fischer Verlag, Stuttgart, pp 63-64, 1992.

113. WEISS HR: Influence of an in-patient exercise program on scoliotic curve. *Italian Journal of Orthopaedics and Traumatology* 18: 395-406, 1992.

114. WEISS HR, WEISS G, SCHAAR HJ: Incidence of surgery in conservatively treated patients with scoliosis. *Pediatric Rehabilitation,* 6: 111-8, 2003.

115. RIGO M, REITER CH, WEISS HR: Effect of conservative management on the prevalence of surgery in patients with adolescent idiopathic scoliosis. *Pediatric Rehabilitation,* 6: 209-214, 2003.

116. WEISS HR, KLEIN R: Improving Excellence in Scoliosis Rehabilitation – A Controlled Study of Matched Pairs. *Pediatric Rehabilitation,* 9 (3): 190-200, 2006.

117. WEISS HR: Bad Sobernheim Concept. Instructional Course Lecture. 3rd International Conference on Conservative Management of Spinal Deformities. Poznan, Poland, 7-8 April 2006

118. DOBOSIEWICZ, K, DURMAŁA J, CZERNICKI K, PIOTROWSKI J: Radiological results of Dobosiewicz method of three-dimensional treatment of progressive Idiopathic Scoliosis. *Stud Health Technol Inform,* 123: 267-272, 2006.

119. NEGRINI S, NEGRINI A, ROMANO M,VERZINI N, PARZINI S: A controlled prospective study on SEAS.02 exercises for idiopathic scoliosis. 3rd International Conference on Conservative Management of Spinal Deformities. Poznan, Poland, 7-8 April 2006.

120. NEGRINI S, NEGRINI A, ROMANO M,VERZINI N, PARZINI S, MONTICONE M: A blind radiographic controlled study on the efficacy of Active Self-Correction according to SEAS.02. 3rd International Conference on Conservative Management of Spinal Deformities. Poznan, Poland, 7-8 April 2006.

121. WEISS HR: Physical Therapy Intervention Studies on Idiopathic Scoliosis - Review with the focus on Inclusion Criteria, *Scoliosis,* 7: in press, 2012.
