# Supplementary material for: Scoliosis short-term rehabilitation (SSTR) according to 'Best Practice' standards - are the results repeatable?
Source: Scoliosis. 2012 Jan 17;7:1. doi: 10.1186/1748-7161-7-1 (PMC3292465; doi:10.1186/1748-7161-7-1)
Supplement: Additional file 4 — Table with the individual results obtained for each single patient (raw data of each patient). [file 1748-7161-7-1-S4.PDF]

Pat. Nr.	Age		Cobb (°)		Curve pattern	ATR (°) thoracic		ATR (°) lumbar		VC in ml	
		Th	*Thl*	*L*		*Before PT*	*After PT*	*Before PT*	*After PT*	*Before PT*	*After PT*
1	15	40		28	4c	17	12/7*	6	6	2600	2900
2	16	34		24	4c	15	11/7*	5	2	2500	2800
3	12	23		14	4c	8	4/2*	6	2	1500	1900
4	13	27		25	4c	12	9/6*	7	3	2200	2500
5	17	30		35	4c	10	6/3*	8	5	2700	3000
6	12	22			3ch	11	7/5*			1900	2100
7	16		35		3ctl	12	8/5*	4	2	2200	2400
8	15	39		21	4c	15	10/7*	5	2	2100	2400
9	15			18	4cl			6	2	2600	2900
10	12		26		4ctl			8	3	1600	1900
11	15	29		13	4c	12	8/6*	4	2	2200	2400
12	16	40		34	4c	16	13/10*	7	5	2800	3100
13	13	33			3ch	13	10/7*			2000	2200
14	12	20			3c	11	8/6*			1500	1700
15	15	34		29	4c	15	12/10*	7	6	2000	2300
16	14	26		22	4c	11	8/6*	5	3	2300	2500
17	11	18			3ch	9	7/5*			1100	1400
18	12	22		19	4c	10	8/6*	5	2	1600	1900
19	14	23			3ch	10	7/5*			2300	2400
20	12	36		24	4c	14	12/9*	6	4	1400	1600
21	16	27			3ch	13	10/8*			2700	2800
22	12	24			3c	12	10/8*			1500	1800
23	16	36			3c	15	11/8*			2800	3000
24	13	28		26	4c	12	9/6*	7	5	1900	2100
25	14	34			3ch	13	10/8*			2200	2400
26	12	22		21	4c	6	5/4*	4	3	1600	1900
27	13	29			3ch	14	11/9*			2100	2400
28	14	32			3ch	12	10/8*			2300	2400
29	13	24		26	4c	7	5/3*	6	3	2300	2600
30	15	43		37	4c	17	14/12*	9	6	2400	2700
31	13			21	4cl			7	3	2200	2400
32	12	21		16	4c	6	4/2*	4	2	1400	1600
33	12	22		27	4c	10	8/6*	8	6	1600	1800
34	14	26			3c	11	9/6*			2600	2900
